# Localization and dynamics of *Wolbachia* infection in Asian citrus psyllid *Diaphorina citri*, the insect vector of the causal pathogens of Huanglongbing

**DOI:** 10.1002/mbo3.561

**Published:** 2018-03-23

**Authors:** Su‐Li Ren, Yi‐Han Li, Da Ou, Yan‐Jun Guo, Jawwad A. Qureshi, Philip A. Stansly, Bao‐Li Qiu

**Affiliations:** ^1^ Key Laboratory of Bio‐Pesticide Innovation and Application Engineering Technology Research Center of Agricultural Pest Biocontrol South China Agricultural University Guangzhou China; ^2^ Airport Management College Guangzhou Civil Aviation College Guangzhou China; ^3^ Institute of Fruit Science Zhaoqing University Zhaoqing China; ^4^ Entomology and Nematology Department University of Florida/IFAS Indian River Research & Education Center Fort Pierce FL USA; ^5^ Southwest Florida Research & Education Center University of Florida/IFAS Immokalee FL USA

**Keywords:** Asian citrus psyllid, endosymbiont, infection dynamic, localization, *Wolbachia*

## Abstract

*Wolbachia* is a group of intracellular bacteria that infect a wide range of arthropods including the Asian citrus psyllid (ACP), *Diaphorina citri* Kuwayama. This insect is the vector of *Candidatus* Liberibacter asiaticus (CLas), the causal pathogen of Huanglongbing or citrus greening disease. Here, we investigated the localization pattern and infection dynamics of *Wolbachia* in different developmental stages of ACP. Results revealed that all developmental stages of ACP including egg, 1st–5th instar nymphs, and adults of both gender were infected with *Wolbachia*. FISH visualization of an ACP egg showed that *Wolbachia* moved from the egg stalk of newly laid eggs to a randomly distributed pattern throughout the egg prior to hatching. The infection rate varied between nymphal instars. The titers of *Wolbachia* in fourth and fifth instar nymphs were significantly higher than those in the first and second instar nymphs. *Wolbachia* were scattered in all nymphal stages, but with highest intensity in the *U*‐shaped bacteriome located in the abdomen of the nymph. *Wolbachia* was confined to two symmetrical organizations in the abdomen of newly emerged female and male adults. The potential mechanisms of *Wolbachia* infection dynamics are discussed.

## 1 INTRODUCTION

Bacterial endosymbionts are widespread microorganisms that are found in many invertebrates including insects, spiders, mites, isopod crustaceans, and filarial nematodes (O'Neill, Giordano, Colbert, Karr, & Robertson, 1992; Pietri, DeBruhl, & Sullivan, 2016; Weeks, Velten, & Stouthamer, 2003; Weinert, Araujo‐Jnr, Ahmed, & Welch, 2015; Zchori‐Fein & Perlman, 2004; Zug & Hammerstein, 2012). Obligate, primary endosymbionts such as *Buchnera* in aphids, *Portiera* in whiteflies and *Uzinura diaspidicola* in armored scales can provide nutrients to these insects that live on a nutritionally unbalanced diet of plant sap during their lifetime (Baumann, 2005; Gruwell, Flarhety, & Dittmar, 2012; Nakabachi et al., 2005). These symbionts are harbored in germ cells of their insect hosts and are vertically transmitted (Baumann, 2005; Weinert et al., 2015). Facultative, secondary endosymbionts are usually dispensable for survival of their hosts, but they can play important roles in manipulating host reproduction in ways that enhance vertical transmission, as well as host fitness, and host defense against thermal stress, natural enemies or pathogens (Brumin, Kontsedalov, & Ghanim, 2011; Hosokawa, Kikuchi, Shimada, & Fukatsu, 2007; Montllor, Maxmen, & Purcell, 2002; Oliver, Moran, & Hunter, 2005; Oliver, Russell, Moran, & Hunter, 2003; Oliver, Smith, & Russell, 2014). Primary symbionts are generally localized in the special cells called bacteriocytes grouped together in a bacteriome, while secondary symbionts have been reported in diverse insect tissues including the brain (Min & Benzer, 1997), salivary glands (Macaluso, Pornwiroon, Popov, & Foil, 2008) malpighian tubules (Bution, Caetano, & Zara, 2008) and hemolymph (Braquart‐Varnier et al., 2008; Fukatsu, Tsuchida, Nikoh, & Koga, 2001).

The Asian citrus psyllid (ACP), *Diaphorina citri* (Hemiptera: Liviidae), is a serious agricultural sap‐sucking pest in citrus‐growing regions of the world. ACP transmits *Candidatus* Liberibacter asiaticus (CLas) bacteria, the causal agent of Huanglongbing (HLB) also known as citrus greening disease (Grafton‐Cardwell, Stelinski, & Stansly, 2013). In addition, feeding and honeydew production of *D. citri* can result in reduced photosynthesis, growth of sooty mold and the death of young foliage at high population densities (Gottwald, 2010; Halbert & Manjunath, 2004). CLas is a phloem‐limited fastidious bacterium, which has not yet been cultured in vitro (Duan et al., 2009; Halbert & Manjunath, 2004). Typical symptoms of HLB include small and bitter fruits; chorotic shoots, blotchy mottle or variegated type of chlorosis, poor root growth, twig dieback and ultimately plant death (Bove, 2006; Gottwald, 2010; Yang et al., 2006).

Two distinct intracellular symbionts are harbored in the yellow and bilobed bacteriome located in the psyllid abdomen. The primary endosymbiont, *Candidatus* Carsonella ruddii, is located in uninucleate bacteriocytes on the surface of the bacteriome, while *Candidatus* Profftella armatura is found in syncytial cytoplasm at the center of the bacteriome (Nakabachi et al., 2013). Besides these primary symbionts, citrus psyllids also harbor secondary symbionts including *Wolbachia* and *Arsenophonus* (Chu, Gill, Hoffmann, & Pelz‐Stelinski, 2016; Saha et al., 2012).

Asian citrus psyllid is the primary vector of *Candidatus* Liberibacter asiaticus in Asia and North America (Gottwald, 2010; Halbert & Manjunath, 2004; Yang et al., 2006). There is no method of cure for HLB‐infected plants (Lopes, Frare, Yamamoto, Ayres, & Barbosa, 2007). Thus, there is an urgent need for effective means to manage the insect vector in order to reduce the incidence of this disease. Symbionts have been considered as a potential approach for control of many insect pests (Benlarbi & Ready, 2003; Mcmeniman et al., 2009; Moreira et al., 2009; Zabalou et al., 2004). Among the secondary endosymbionts, *Wolbachia* is the most abundant in arthropods (Weinert et al., 2015). It can induce reproductive disorders, cytoplasmic incompatibility (CI), parthenogenesis, male feminization and death; all of which warrant their manipulation as potential control agents (O'Neill et al., 1997; Werren, 1997; Werren, Baldo, & Clark, 2008) with cytoplasmic incompatibility being the most promising. This favors a particular *Wolbachia* strain that induces early embryonic death to egg and sperm combinations that are not both infected with the same strain. The potential use of this mechanism to control mosquitos has been explored in several studies including Xi and Dobson (2005), Kambris, Cook, Phuc, and Sinkins (2009), Moreira et al. (2009), Bian, Xu, Lu, Xie, and Xi (2010) and Walker et al. (2011). In addition, *Wolbachia* strains such as *wMel*,* wAlbB* have been used to suppress transmission of human pathogens in *Anopheles gambiae*,* A. stephensi* and *Aedes albopictus,* respectively (Bian et al., 2013; Blagrove, Arias‐Goeta, Failloux, & Sinkins, 2012; Hughes, Koga, Xue, Fukatsu, & Rasgon, 2011). It is therefore likely that endosymbionts, such as *Wolbachia* could be used to manipulate reproduction of ACP through cytoplasmic incompatibility and so suppress transmission of CLas to citrus plants (Hoffmann, Coy, Gibbard, & Pelz‐Stelinski, 2014). However, to achieve this goal it is essential to understand the infection biology of *Wolbachia* in ACP, including determining the identity of the strains, their infection level and localization patterns (Chu et al., 2016; Kruse et al., 2017).

In this study, we used PCR, qPCR, and whole‐mount fluorescence in situ hybridization (wFISH) to firstly detect the infection prevalence of *Wolbachia*, and secondly, determine the localization pattern of this endosymbiont in all life stages of ACP.

## 2 MATERIAL AND METHODS

### 2.1 Insects

The Asian citrus psyllid population used in this study was collected in September 2013, from healthy *Murraya exotica* L. (Rutaceae) plants on the campus of South China Agricultural University (SCAU, 23°09′N, 113°20′E), Guangzhou city, China. The psyllids were then reared for several generations on young *M. exotica* plants in a greenhouse in SCAU under ambient temperature and photoperiod before experiments were initiated.

### 2.2 DNA extraction from ACP

To extract the DNA, eggs, nymphs, and adults of both genders were collected from *M. exotica* plants, washed with 70% ethanol and then dried at room temperature. Nymphs were separated by instar based on their morphological characteristics (Tsai & Liu, 2000).

DNA extractions were conducted by two methods. In the first method, individual psyllids were first washed with double distilled water to remove all alcohol. The sample containing either one individual of each nymphal instar, a male or female adult, or 10 eggs together as one unit due to their small size was homogenized in 2μl STE (10 mmol/L Tris‐HCl, pH 8.0, 25 mmol/L NaCl, 25 mmol/L EDTA, 1% SDS, proteinase K 200 mg/ml) in a 0.5 ml microcentrifuge tube. The mixture for each sample was finally complemented with 15 μl STE in the 0.5 ml microcentrifuge tube. The homogenate was incubated at 56°C for 2–3 hr and then placed in 95°C water for 10 min to inactivate the proteinase K. After incubation, the samples were centrifuged for a short time and then used for PCR detection of *Wolbachia*.

In the second method, total DNA was extracted from groups of 40–50 ACP eggs, 1–2 instar nymphs or 10–20 individuals of 3–5 instar nymphs, male/female adults for qPCR using the TIANamp genomic DNA kit (TIANGEN Biotech, Beijing, China) with minor modifications for preparation of DNA from animal tissues. To assess DNA integrity, each sample was separated by electrophoresis on a 1% agarose gel (1%, 0.05μl/ml GoldView, TRIS‐EDTA‐Buffer) at 5 V/cm, and visualized on a UV transilluminator and then photographed via the gel imager. Additionally, quality and quantity of total DNA was measured on a NanoDrop 2,000 spectrophotometer to ensure uniformity among all samples for qPCR (Dossi, Da Silva, & Consoli, 2014; Tiwari, Gondhalekar, Mann, Scharf, & Stelinski, 2011).

### 2.3 PCR detection of *Wolbachia* in ACP

PCR detection of *Wolbachia* was conducted in a 25 μl reaction volume containing: 16 μl of double distilled water, 6 μl of 2xHiFiTaq PCR starMix Genstar, Beijing, China, 1 μl of each primer solution (20 μmol/L each), and 1 μl of DNA template of each ACP sample (egg, 1st–5th instar nymph, male or female adult). The primers were wsp, 81F: 5′‐TGGTCCAATAAGTGATGA AGAAAC‐3′, 691R: 5′‐AAAAATT AAACGCTACTCCA‐3′, which are specific to the *Wolbachia* endosymbiont (Braig, Zhou, Dobson, & O'Neill, 1998). The PCR procedure was: pre‐denatured at 94°C for 3 min, followed by 35 cycles at 94°C for 35 s, 55°C for 30 s, 72°C for 30 s, and a final extension at 72°C for 10 min. PCR amplified products were visualized on a 1% agarose gel containing GoldView colorant. When bands with the expected size were visible in the gels, 20 μl PCR products were sent for sequencing.

As mentioned above, ten eggs together as a unit, one individual of 1st–5th instar nymph, or one adult of each gender were treated as one replicate. In total 30 replicates were tested (10 replicates in one repeat ×3) in each experiment. Each PCR reaction included a positive (primary endosymbiont, *Carsonella*) and negative (ddH_2_O) control to verify DNA quality.

### 2.4 Quantification of *Wolbachia* titer in different life stages of ACP

*Wolbachia* was quantified by the SYBR Premix Ex Taq in the CFX‐96 Real‐Time PCR system (Bio‐Rad). The primers for qPCR were the wsp gene specific for *Wolbachia*: wsp‐F: 5′‐TGGTCCAATA AGTGATGAAGAAAC‐3′, wsp‐R: 5′‐AAAAATTAAACGCTACTCCA‐3′ (Ghanim & Kontsedalov, 2009). One β‐actin gene from ACP itself was used as an internal standard for data normalization. The primers of β‐actin were F: 5′‐CCCTGGACTTTGA ACAGGAA‐3′, β‐actin R: 5′‐CTCGTGGATACCGC AAGATT‐3′ (Tiwari et al., 2011). The qPCR reaction was a 25 μl volume containing: 12.5 μl of SYBR Premix Ex Taq (TIANGEN Biotech, Beijing, China), 9.5 μl of RNase‐free water, 0.5 μl of each primer solution (10 μmol/L each), and 2 μl of DNA template for each ACP sample. The qPCR procedure was initiated with 5‐min activation at 95°C followed by 40 cycles of 10 s at 95°C, 30 s at 60°C, and 60 s at 72°C. Again, ten eggs as a unit, one individual of 1st–5th instar nymph, or one male/female adult were detected as one replicate. In total four replicates for each developmental stage were repeated in this qPCR analysis.

### 2.5 Distribution of *Wolbachia* in different life stages of ACP

Eggs, and nymphs of each instar stage along with newly eclosed adults of ACP were collected from healthy *M. exotica* shoots with a camel‐hair brush. Fluorescence in situ hybridization (FISH) analysis of different psyllids stages and gender was performed as described by Gottlieb et al. (2006) with the probe W2‐Cy3 (5′‐Cy3‐CTTCTGTGAGTACCGTCATTATC‐3′) in order to detect *Wolbachia*. The samples were whole mounted, stained, and observed using an inverted fluorescence microscope (Nikon Eclipse Ti‐U). For each sample, at least 50 specimens were examined to confirm the results. *Wolbachia* infected ACPs (from the *Wolbachia* positive population) with no FISH probe were used as a control to confirm the specificity of *Wolbachia* detection.

### 2.6 Statistical analysis

Differences among nymphal stages and between male and female adult ACP in incidence and titer of *Wolbachia* were analyzed using one‐way ANOVA (SPSS 17.0 software, SPSS Inc., Chicago, IL, USA). Fisher's protected Duncan test was used for mean separation contingent on a significant treatment *F* value.

## 3 RESULTS

### 3.1 PCR detection of *Wolbachia* in ACP

**Figure 1:**
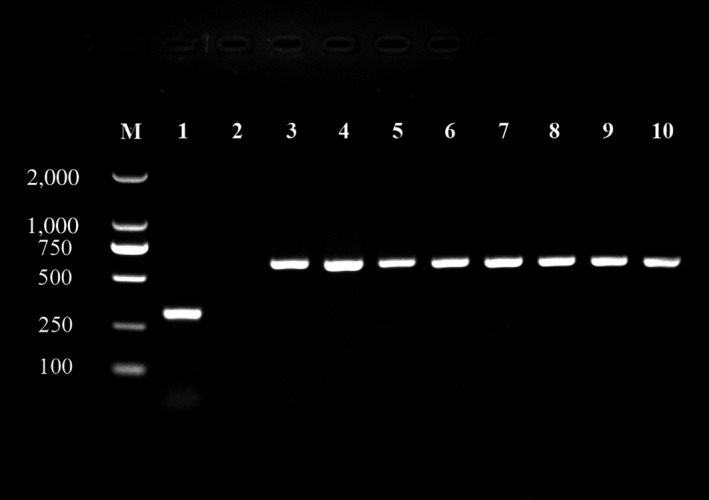
*Wolbachia* detection in different developmental stages of Asian citrus psyllid using PCR. Lane M: DL2,000 marker; Lane (1–10): positive control *Carsonella*, ddH_2_O negative control, male adult, female adult, fifth instar nymph, fourth instar nymph, third instar nymph, second instar nymph, first instar nymph, egg

*Wolbachia* wsp specific DNA was detected by PCR in all life stages of ACP including egg, nymphs, and adults (Figure 1). However, the infection rates of *Wolbachia* varied somewhat among different stages: 90.0 ± 5.8% in eggs, 90%–100% among 1st–5th instar nymphs, 96.7% ± 3.3% in adult females and 100% in adult males (*N* = 30). However, these differences were not significantly different (Table 1).

**Table 1:** The infection rates of *Wolbachia* in different developmental stages and genders of Asian citrus psyllid

Stages	Total individuals	Positive individuals	*Wolbachia* infection rate (%)
Egg^a^	30	27	90.00 ± 5.77a^b^
1st instar	30	27	90.00 ± 5.77a
2nd instar	30	30	100.00 ± 0a
3rd instar	30	29	96.67 ± 3.33a
4th instar	30	29	96.67 ± 3.33a
5th instar	30	28	93.33 ± 3.33a
Male adult	30	30	100.00 ± 0a
Female adult	30	29	96.67 ± 3.33a

a Each individual sample contained 10 eggs.

b the same letter in one volume means no significant differences between each other at *p* < 0.05 (Duncan test).

### 3.2 Quantification of *Wolbachia* titer in different stages of ACP

**Figure 2:**
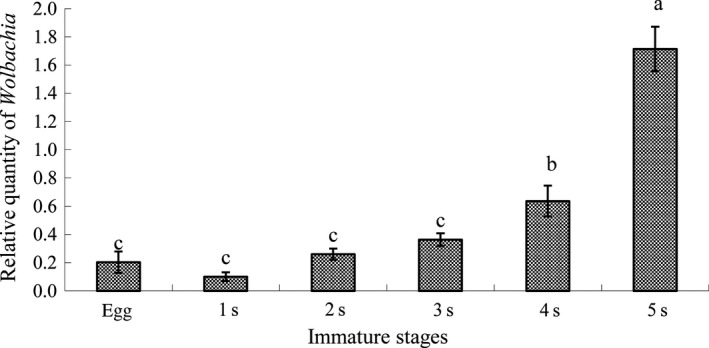
Relative quantity (mean ± SE) of *Wolbachia* in egg and nymphal instars of Asian citrus psyllid calculated using the method of 2^−ΔΔct^. Columns with the same letter represent means with no significant difference at *p* < .05

**Figure 3:**
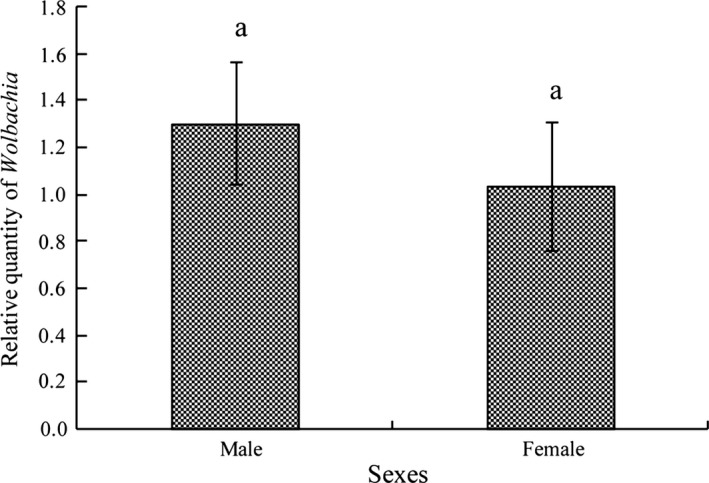
Relative quantity (mean ± SE) of *Wolbachia* in male and female adults of Asian citrus psyllid calculated using the method of 2^−ΔΔct^. No significant difference between gender

Taking the psyllid actin gene as the baseline, the titer of *Wolbachia* increased with successive nymphal instars (Figure 2), for example, *Wolbachia* titers in the 4th–5th instar nymphs were significantly higher than those in the 1st–3rd instar nymphs (*F* = 45.37, *p* < .0001). The *Wolbachia* titer of 5th instar ACP nymph was even higher than that of the ACP male and female adults, but no significant differences were found between the nymph and adults. One interesting finding was that, the titer of *Wolbachia* in ACP eggs was higher than that of the first instar nymph. The titer of *Wolbachia* did not differ significantly between adult genders but was relatively higher in males than in females (*F* = 0.51, *p* = .5007, Figure 3).

### 3.3 Distribution of *Wolbachia* in different life stages of ACP using Fluorescence in situ hybridization

**Figure 4:**
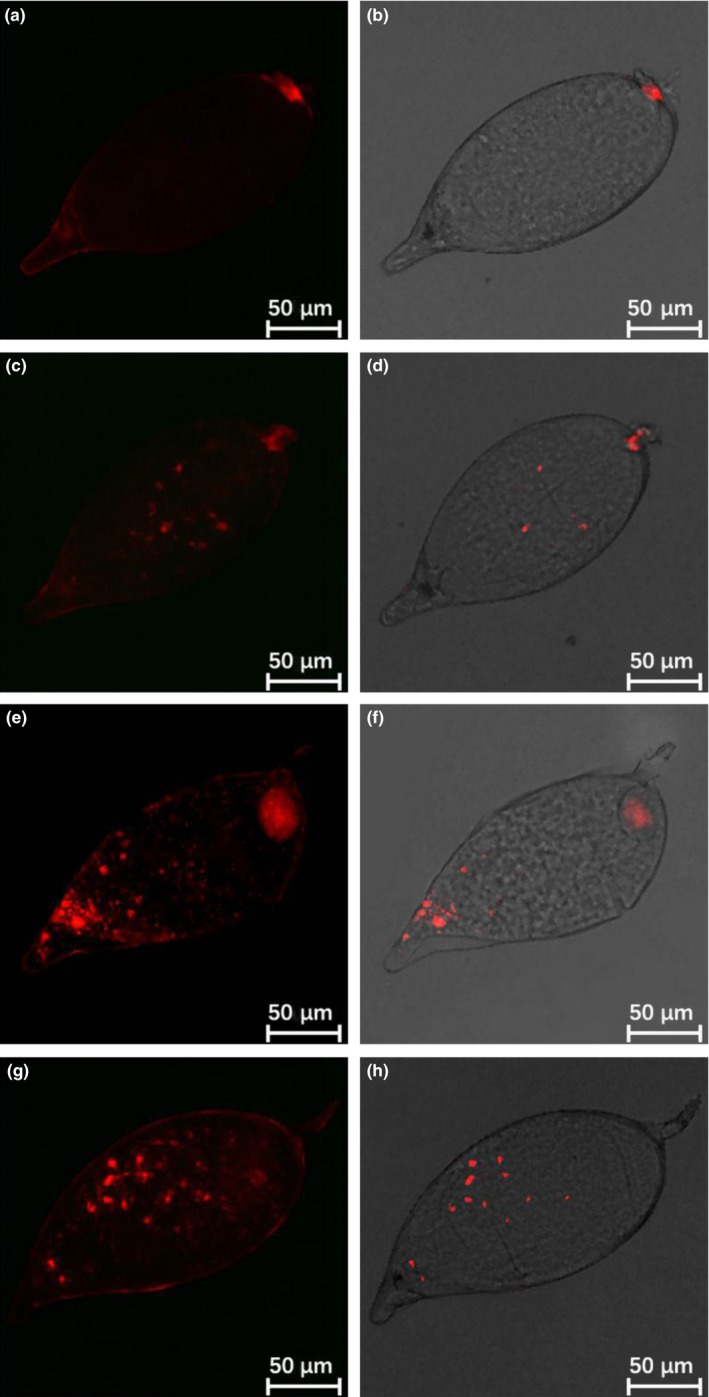
FISH visualization of *Wolbachia* during egg stage of Asian citrus psyllid. (a and b) 0–1 day old eggs; (c and d) 1–2 day old eggs; (e and f) 2–3 day old eggs; (g and h) 3–4 day old eggs. Left panels: fluorescence in dark field; right panels: fluorescence in bright field

Distribution of *Wolbachia* varied over the course of egg development. *Wolbachia* was most concentrated in the bacteriome at the basal pedicel end of newly laid eggs, although a more diffuse concentration could also be seen around the apex (Figure 4a and b). Later on, *Wolbachia* gradually spread out from the two poles to give a more uniform distribution (Figure 4c and f). In older eggs, *Wolbachia* were more random in distribution (Figure 4g and h). Incidence of *Wolbachia* over all egg specimens was 90.9% (40/44) as determined by FISH visualization detection.

**Figure 5:**
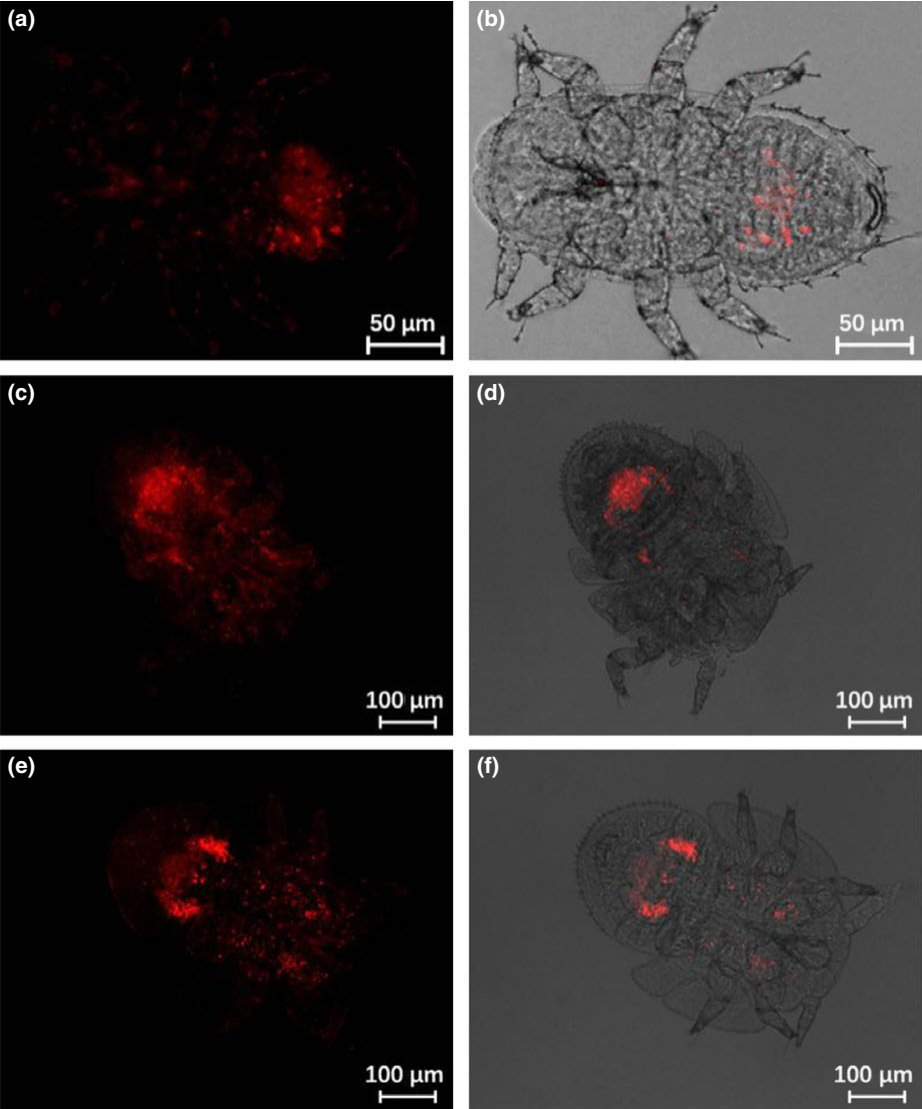
FISH visualization of *Wolbachia* in young‐instar nymphs of Asian citrus psyllid. (a and b) first instar nymphs; (c and d) second instar nymphs; (e and f) third instar nymphs. Left panels: fluorescence in dark field; right panels: fluorescence in bright field

**Figure 6:**
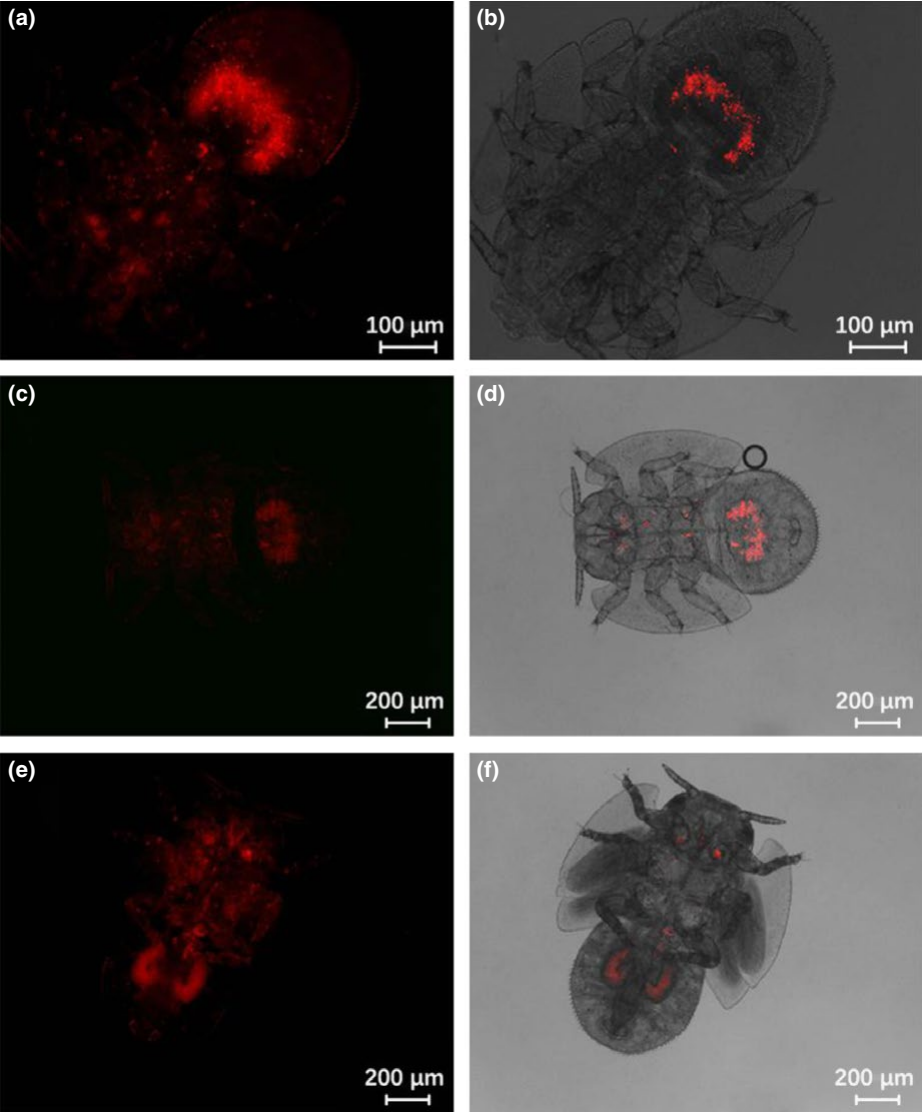
FISH visualization of *Wolbachia* in mature‐instar nymphs of Asian citrus psyllid. (a and b) fourth instar nymphs; (c and d) fifth instar nymphs; (e‐f) the end of fifth instar nymphs. Left panels: fluorescence in dark field; right panels: fluorescence in bright field

**Figure 7:**
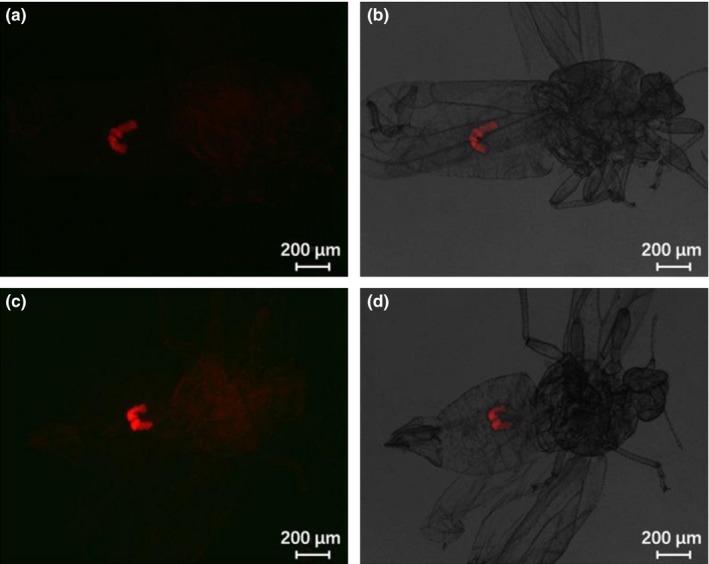
FISH visualization of *Wolbachia* in male and female adults of Asian citrus psyllid. (a and b) female adults; (c and d) male adults. Left panels: fluorescence in dark field; right panels: fluorescence in bright field

*Wolbachia* localized primarily in the abdomen of ACP nymphs. The FISH signal could be detected throughout the nymph, but at highest intensity in the U‐shaped bacteriome in the nymphal abdomen (Figures 5 and 6). Incidence of *Wolbachia* infection over all nymphs examined by FISH was 78.6% (55/70). Incidence in adults was similar to nymphs at 76.2% (16/21). The symbionts occupied two symmetrical organizations in the adult abdomen thought to be the group of bacteriomes (Figure 7).

## 4 DISCUSSION

Numerous studies have revealed the biological roles of endosymbionts in the development, reproduction and defense of their insect hosts (Dale & Moran, 2006; Oliver, Degnan, Burke, & Moran, 2010; Siozios, Sapountzis, Ioannidis, & Bourtzis, 2008; Zug & Hammerstein, 2015a). Among these, *Wolbachia* are intracellular bacteria that infect a vast range of arthropod species, making them one of the most abundant endosymbionts in nature. The stunning evolutionary success of *Wolbachia* is mostly due to their reproductive parasitism but also mutualistic effects such as increased host fecundity and protection against pathogens (Zug & Hammerstein, 2015b). In the current study, detection frequencies of *Wolbachia* in ACP varied among different life stages and between gender from 100% in both the second instar nymphs and adult males to 90.0% in eggs and first instar nymphs. Guidolin and Consoli (2013) reported 100% incidence of *Wolbachia* in ACP specimens tested in Brazil. Subandiyah, Nikoh, Tsuyumu, Somowiyarjo, and Fukatsu (2000) found *Wolbachia* in 76.2% of *D. citri* adults sampled in Japan. Some differences in *Wolbachia* infection rates may result from geographic variation, number of ACP sampled and the methods used for detection. Furthermore, the infection of *Wolbachia* in ACPs was detected by three methods, normal PCR, FISH and qPCR in this study; the revealed infection rates were around 90%–100% by normal PCR, 77%–79% by FISH and 100% by qPCR, which indicated that there were certain differences among the three methods with qPCR being the most sensitive and accurate.

We found that the infection titer of *Wolbachia* tended to increase with successive nymphal instars in concert with developmental time. This result agreed with a recent study of Dossi et al. (2014), which reported an increase in *Wolbachia* densities with development of ACP populations in Brazil. Both studies found that *Wolbachia* titer was greater in the late embryonic egg stage of ACP compared with the first instar nymph. We deduce that this may due to two reasons: firstly, *Wolbachia* is mostly maternally transmitted from female to offspring, therefore, ovaries and mature eggs usually harbor more *Wolbachia* than other tissues, however, after egg hatching *Wolbachia* probably get scattered, reducing in numbers due to spreading into newly developing tissues; secondly, it might not be able to adapt to the new immune system which starts after the stage change from egg to nymph in order to regulate the related host immunity (Douglas, Bouvaine, & Russell, 2011; Gorman, Kankanala, & Kanost, 2004; Nishikori, Morioka, Kubo, & Morioka, 2009). However, regardless of both possibilities, the causation of the mechanism of infection warrants further study. Other studies have shown that environmental changes, such as insecticide exposure, temperature, host genotype diversity and *Wolbachia* strain can also influence their titer (Hurst, Jiggins, & Robinson, 2001; Weeks, Reynolds, Hoffmann, & Mann, 2002).

Our finding that more *Wolbachia* is present in males than females is consistent with previous related work with ACP (Hoffmann et al., 2014) as well as the pattern of *wAlbB* infection in *Aedes albopictus* (Tortosa et al., 2010). In many other insect species, the titer of *Wolbachia* is usually higher in adult females than in males (Correa & Ballard, 2012; Mouton et al., 2004; Tortosa et al., 2010). However, the reasons for the higher titers of *Wolbachia* in male compared to female ACP are still not clear. Rio, Wu, Filardo, and Aksoy (2006) found that the *Wolbachia* density in male tsetse fly was much higher than that of female, with males also showing a broader tissue distribution of *Wolbachia* compared to females. They deduced that there might be a sex specific opportunist role for *Wolbachia* replication in males, or that there exists a density regulation that is mediated by the insect host or a symbiont in the female, whereas this regulation efficacy is lost in males. Dossi et al. (2014) supposed that the lower density of *Wolbachia* in older females could be a consequence of the reduced growth rate of third instar due to the process of transovarian transmission. Moreover, the difference of *Wolbachia* titers between ACP male and females maybe also related to their Las infection status. Fagen et al. (2012) observed a strong positive correlation between *Wolbachia* and CLas titers within ACP, Kolora, Powell, Hunter, Bextine, and Lauzon (2015) also found that the amount of *Wolbachia* in ACP was greater in insects infected with CLas, whereas Chu et al. (2016) revealed that both the densities of primary *Carsonella* and facultative *Wolbachia* were significantly higher in CLas negative ACP compared to CLas‐positive ACP Florida populations. Therefore, to reveal which gender has a higher capability to harbor and transmit CLas may assist in further understanding the complicated association among CLas, *Wolbachia*, different gender of ACP as well as different populations or genotypes of ACP.

In this study, we were able to pinpoint the dynamics of localization patterns of *Wolbachia* in ACP using the whole‐mount fluorescence in situ hybridization method. Our FISH results revealed an uneven distribution pattern of *Wolbachia* in most of the ACP eggs and nymphal stages. Migration of *Wolbachia* from the egg stalk toward the central egg region is reminiscent of displacement of *Rickettsia* in *Bemisia tabaci* eggs (Gottlieb et al., 2006). Localization of *Wolbachia* in different parts of the egg appears to be related to diversion of the cytoskeleton which is known to play an essential role in repartition of organelles in cells (Sicard, Dittmer, Greve, Bouchon, & Braquart‐Varnier, 2014).

In nymphs, we found the highest concentration of *Wolbachia* in the U‐shaped bacteriome located in the abdomen, with lower concentrations in the thorax, and occasional presence in the head. The ACP bacteriome is known to harbor three symbionts: *Carsonella*,* Profftella* (Nakabachi et al., 2013), and now *Wolbachia*. This result suggests a specific immune profile for *Wolbachia* allowing the host to maintain and control the symbiosis (Anselme, Vallier, Balmand, Fauvarque, & Heddi, 2006; Heddi et al., 2005). The distribution of *Wolbachia* in late fifth instar nymphs is quite similar to that in ACP adults; reflecting the transition from nymph to adult. Using FISH methodology, Kruse et al. (2017) found that *Wolbachia* has a widespread distribution throughout the ACP gut tissue, including the midgut, filter chamber and Malpighian tubules. They also determined that *Wolbachia* and CLas are capable of residing in the same ACP gut cells, but that they do not have a high degree of co‐localization within cells.

The localization of *Wolbachia* has also been studied in other insects. In the bedbug *Cimex lectularius*,* Wolbachia* symbiont was specifically localized in the bacteriomes and vertically transmitted via the somatic stem cell niche of germalia to oocytes. Here, it infected the incipient symbiotic organ at an early stage of the embryogenesis in adults. In the males, *Wolbachia* was restricted to the testis‐associated bacteriomes, whereas in the females, it was found in the bacteriomes and the ovaries (Dobson et al., 1999). In *Drosophila melanogaster*, Clark, Veneti, Bourtzis, and Karr (2002, 2003) determined that *Wolbachia* were found inside spermatocytes and spermatids or within the somatic cyst cells surrounding the germ cells, and throughout development there appeared little movement of *Wolbachia* between spermatids via the connecting cytoplasmic bridges. In the endosymbiont‐scale insect system, Gruwell et al. (2012) found that the endosymbiont *Uzinura diaspidicola* localized in all the developmental stages of armored scale insects, including embryos, eggs and adults, which is similar to the findings in this study. All of these studies indicate a close association between *Wolbachia* endosymbiont and its insect host development, indicating the potential to develop novel approaches for managing citrus HLB, such as prevention of CLas transmission from the endosymbiont viewpoint.

The potential of *Wolbachia* to control disease vectors, and interfere with the ability of mosquitos to vector malaria and dengue has been demonstrated (Bian et al., 2013; Bourtzis et al., 2014; Guidolin & Consoli, 2013). As mentioned above, Fagen et al. (2012) and Kolora et al. (2015) reported that *Wolbachia* has a positive association with the CLas, while Chu et al. (2016) revealed that both the densities of primary *Carsonella* and facultative *Wolbachia* were significantly higher in CLas‐negative ACP compared to CLas‐positive ACP. Whichever reflect the true infection status in the field, the interactions of *Wolbachia*‐CLas can be further explored as a novel strategy to potentially control HLB through artificial manipulation of insect symbionts. Moreover, our molecular phylogenetic study has indicated that the *Wolbachia* of ACP from South China belongs to the *Con* strain in the *Wolbachia* B supergroup. The potential strategy of using *Wolbachia* to reduce ACP populations in the field may be practical by releasing a male ACP population with another strain of *Wolbachia* (single strain strategy, to realize this work we can first eliminate the original strain of *Wolbachia* and infect the ACP with a new strain by artificial micro‐infection), or overlay with another strain with this *Con* strain (double strain strategy). Therefore, cytoplasmic incompatibility may occur when these two types of male adults mate with wild female ACP adults.

In summary, considering the potential use of *Wolbachia* for vector and disease management, studies on the ecological factors that affect the interactions between *Wolbachia* and its ACP host may be beneficial in developing novel strategies for ACP and HLB management. The current study moves toward this final goal.

## CONFLICT OF INTEREST

None declared.

## References

[mbo3561-bib-0001] Anselme, C. , Vallier, A. , Balmand, S. , Fauvarque, M. O. , & Heddi, A. (2006). Host PGRP gene expression and bacterial release in endosymbiosis of the weevil *Sitophilus zeamais* . Applied and Environmental Microbiology, 72, 6766–6772. 10.1128/AEM.00942-06 17021229 PMC1610295

[mbo3561-bib-0002] Baumann, P. (2005). Biology of bacteriocyte‐associated endosymbionts of plant sap‐sucking insects. Annual Review of Microbiology, 59, 155–189. 10.1146/annurev.micro.59.030804.121041 16153167

[mbo3561-bib-0003] Benlarbi, M. , & Ready, P. D. (2003). Host‐specific *Wolbachia* strains in widespread populations of *Phlebotomus perniciosus* and *P. papatasi* (Diptera: Psychodidae), and prospects for driving genes into these vectors of Leishmania. Bulletin of Entomological Research, 93, 383–391. 14641977 10.1079/ber2003251

[mbo3561-bib-0004] Bian, G. W. , Joshi, D. , Dong, Y. M. , Lu, P. , Zhou, G. L. , Pan, X. L. , … Xi, Z. Y. (2013). *Wolbachia* invades *Anopheles stephensi* populations and induces refractoriness to *Plasmodium* infection. Science, 340, 748–751. 10.1126/science.1236192 23661760

[mbo3561-bib-0005] Bian, G. W. , Xu, Y. , Lu, P. , Xie, Y. , & Xi, Z. Y. (2010). The endosymbiotic bacterium *Wolbachia* induces resistance to dengue virus in *Aedes aegypti* . PLoS Pathogens, 6, e1000833. 20368968 10.1371/journal.ppat.1000833 PMC2848556

[mbo3561-bib-0006] Blagrove, M. S. C. , Arias‐Goeta, C. , Failloux, A. B. , & Sinkins, S. P. (2012). *Wolbachia* strain wMel induces cytoplasmic incompatibility and blocks dengue transmission in *Aedes albopictus* . Proceedings of the National Academy of Sciences of the United States of America, 109, 255–260. 10.1073/pnas.1112021108 22123944 PMC3252941

[mbo3561-bib-0007] Bourtzis, K. , Dobson, S. L. , Xi, Z. Y. , Rasgon, J. L. , Calvitti, M. , Moreira, L. A. , … Gilles, J. R. L. (2014). Harnessing mosquito‐*Wolbachia* symbiosis for vector and disease control. Acta Tropica, 132, S150–S163. 10.1016/j.actatropica.2013.11.004 24252486

[mbo3561-bib-0008] Bove, J. M. (2006). Huanglongbing: A destructive, newly‐emerging, century‐old disease of citrus. Journal of Plant Pathology, 88, 7–37.

[mbo3561-bib-0009] Braig, H. R. , Zhou, W. , Dobson, S. L. , & O'Neill, S. L. (1998). Cloning and characterization of a gene encoding the major surface protein of the bacterial endosymbiont *Wolbachia pipientis* . Journal of Bacteriology, 180, 2373–2378. 9573188 10.1128/jb.180.9.2373-2378.1998 PMC107178

[mbo3561-bib-0010] Braquart‐Varnier, C. , Lachat, M. , Herbiniere, J. , Johnson, M. , Caubet, Y. , Bouchon, D. , & Sicard, M. (2008). *Wolbachia* mediate variation of host immunocompetence. PLoS ONE, 3, e3286 10.1371/journal.pone.0003286 18818770 PMC2546445

[mbo3561-bib-0011] Brumin, M. , Kontsedalov, S. , & Ghanim, M. (2011). *Rickettsia* influences thermotolerance in the whitefly *Bemisia tabaci* B biotype. Insect Science, 18, 57–66. 10.1111/ins.2011.18.issue-1

[mbo3561-bib-0012] Bution, M. L. , Caetano, F. H. , & Zara, F. J. (2008). Contribution of the Malpighian tubules for the maintenance of symbiotic microorganisms in cephalotes ants. Micron, 39, 1179–1183. 10.1016/j.micron.2008.05.003 18579390

[mbo3561-bib-0013] Chu, C. C. , Gill, T. A. , Hoffmann, M. , & Pelz‐Stelinski, K. S. (2016). Inter‐population variability of endosymbiont densities in the Asian citrus psyllid (*Diaphorina citri* Kuwayama). Microbial Ecology, 71, 999–1007. 10.1007/s00248-016-0733-9 26846216 PMC4944574

[mbo3561-bib-0014] Clark, M. E. , Veneti, Z. , Bourtzis, K. , & Karr, T. L. (2002). The distribution and proliferation of the intracellular bacteria *Wolbachia* during spermatogenesis in *Drosophila* . Mechanisms of Development, 111, 3–15. 10.1016/S0925-4773(01)00594-9 11804774

[mbo3561-bib-0015] Clark, M. E. , Veneti, Z. , Bourtzis, K. , & Karr, T. L. (2003). *Wolbachia* distribution and cytoplasmic incompatibility during sperm development: The cyst as the basic cellular unit of CI expression. Mechanisms of Development, 120, 185–198. 10.1016/S0925-4773(02)00424-0 12559491

[mbo3561-bib-0016] Correa, C. C. , & Ballard, J. W. O. (2012). *Wolbachia* gonadal density in female and male *Drosophila* vary with laboratory adaptation and respond differently to physiological and environmental challenges. Journal of Invertebrate Pathology, 111, 197–204. 10.1016/j.jip.2012.08.003 22903036

[mbo3561-bib-0017] Dale, C. , & Moran, N. A. (2006). Molecular interactions between bacterial symbionts and their hosts. Cell, 126, 453–465. 10.1016/j.cell.2006.07.014 16901780

[mbo3561-bib-0018] Dobson, S. L. , Bourtzis, K. , Braig, H. R. , Jones, B. F. , Zhou, W. , Rousset, F. , & O'Neill, S. L. (1999). *Wolbachia* infections are distributed throughout insect somatic and germ line tissues. Insect Biochemistry and Molecular Biology, 29, 153–160. 10.1016/S0965-1748(98)00119-2 10196738

[mbo3561-bib-0019] Dossi, F. C. A. , Da Silva, E. P. , & Consoli, F. L. (2014). Population dynamics and growth rates of endosymbionts during *Diaphorina citri* (Hemiptera, Liviidae) ontogeny. Microbial Ecology, 68, 881–889. 10.1007/s00248-014-0463-9 25037159

[mbo3561-bib-0020] Douglas, A. E. , Bouvaine, S. , & Russell, R. R. (2011). How the insect immune system interacts with an obligate symbiotic bacterium. Proceedings of the Royal Society B‐Biological Sciences, 278, 333–338. 10.1098/rspb.2010.1563 PMC3013419 20719775

[mbo3561-bib-0021] Duan, Y. P. , Zhou, L. J. , Hall, D. G. , Li, W. B. , Doddapaneni, H. , Lin, H. , … Gottwald, T. (2009). Complete genome sequence of citrus huanglongbing bacterium, ‘*Candidatus* Liberibacter asiaticus’ obtained through metagenomics. Molecular Plant‐Microbe Interactions, 22, 1011–1020. 10.1094/MPMI-22-8-1011 19589076

[mbo3561-bib-0022] Fagen, J. R. , Giongo, A. , Brown, C. T. , Davis‐Richardson, A. G. , Gano, K. A. , & Triplett, E. W. (2012). Characterization of the relative abundance of the citrus pathogen *Ca*. Liberibacter asiaticus in the microbiome of its insect vector, *Diaphorina citri*, using high throughput 16S rRNA sequencing. The Open Microbiology Journal, 6, 29–33. 10.2174/1874285801206010029 22529882 PMC3330398

[mbo3561-bib-0023] Fukatsu, T. , Tsuchida, T. , Nikoh, N. , & Koga, R. (2001). *Spiroplasma* symbiont of the pea aphid, *Acyrthosiphon pisum* (Insecta: Homoptera). Applied and Environmental Microbiology, 67, 1284–1291. 10.1128/AEM.67.3.1284-1291.2001 11229923 PMC92726

[mbo3561-bib-0024] Ghanim, M. , & Kontsedalov, S. (2009). Susceptibility to insecticides in the Q biotype of *Bemisia tabaci* is correlated with bacterial symbiont densities. Pest Management Science, 65, 939–942. 10.1002/ps.v65:9 19479746

[mbo3561-bib-0025] Gorman, M. J. , Kankanala, P. , & Kanost, M. R. (2004). Bacterial challenge stimulates innate immune responses in extra‐embryonic tissues of tobacco hornworm eggs. Insect Molecular Biology, 13, 19–24. 10.1111/imb.2004.13.issue-1 14728663

[mbo3561-bib-0026] Gottlieb, Y. , Ghanim, M. , Chiel, E. , Gerling, D. , Portnoy, V. , Steinberg, S. , … Zchori‐Fein, E. (2006). Identification and localization of a *Rickettsia* sp. in *Bemisia tabaci* (Homoptera: Aleyrodidae). Applied and Environmental Microbiology, 72, 3646–3652. 10.1128/AEM.72.5.3646-3652.2006 16672513 PMC1472322

[mbo3561-bib-0027] Gottwald, T. R. (2010). Current epidemiological understanding of citrus Huanglongbing. Annual Review of Phytopathology, 48, 119–139. 10.1146/annurev-phyto-073009-114418 20415578

[mbo3561-bib-0028] Grafton‐Cardwell, E. E. , Stelinski, L. L. , & Stansly, P. A. (2013). Biology and management of Asian citrus psyllid, vector of the Huanglongbing pathogens. Annual Review of Entomology, 58, 413–432. 10.1146/annurev-ento-120811-153542 23317046

[mbo3561-bib-0029] Gruwell, M. E. , Flarhety, M. , & Dittmar, K. (2012). Distribution of the primary endosymbiont (*Candidatus* Uzinura Diaspidicola) within host insect from the scale insect family Diaspididae. Insects, 3, 262–269. 10.3390/insects3010262 26467959 PMC4553627

[mbo3561-bib-0030] Guidolin, A. S. , & Consoli, F. L. (2013). Molecular characterization of *Wolbachia* strains associated with the invasive Asian citrus psyllid *Diaphorina citri* in Brazil. Microbial Ecology, 65, 475–486. 10.1007/s00248-012-0150-7 23269454

[mbo3561-bib-0031] Halbert, S. E. , & Manjunath, K. L. (2004). Asian citrus psyllids (Sternorrhyncha:Psyllidae) and greening disease of citrus: A literature review and assessment of risk in Florida. Florida Entomologist, 87, 330–353. 10.1653/0015-4040(2004)087[0330:ACPSPA]2.0.CO;2

[mbo3561-bib-0032] Heddi, A. , Vallier, A. , Anselme, C. , Xin, H. , Rahbe, Y. , & Wackers, F. (2005). Molecular and cellular profiles of insect bacteriocytes: Mutualism and harm at the initial evolutionary step of symbiogenesis. Cellular Microbiology, 7, 293–305. 15659072 10.1111/j.1462-5822.2004.00461.x

[mbo3561-bib-0033] Hoffmann, M. , Coy, M. R. , Gibbard, H. N. K. , & Pelz‐Stelinski, K. S. (2014). *Wolbachia* infection density in populations of the Asian citrus psyllid (Hemiptera: Liviidae). Environmental Entomology, 43, 1215–1222. 10.1603/EN14193 25259690

[mbo3561-bib-0034] Hosokawa, T. , Kikuchi, Y. , Shimada, M. , & Fukatsu, T. (2007). Obligate symbiont involved in pest status of host insect. Proceedings of the Royal Society B‐Biological Sciences, 274, 1979–1984. 10.1098/rspb.2007.0620 PMC2275188 17567556

[mbo3561-bib-0036] Hughes, G. L. , Koga, R. , Xue, P. , Fukatsu, T. , & Rasgon, J. L. (2011). *Wolbachia* infections are virulent and inhibit the human malaria parasite *Plasmodium falciparum* in *Anopheles gambiae* . PLoS Pathogens, 7, e1002043 10.1371/journal.ppat.1002043 21625582 PMC3098226

[mbo3561-bib-0037] Hurst, G. D. D. , Jiggins, F. M. , & Robinson, S. J. W. (2001). What causes inefficient transmission of male‐killing *Wolbachia* in *Drosophila*? Heredity, 87, 220–226. 10.1046/j.1365-2540.2001.00917.x 11703513

[mbo3561-bib-0038] Kambris, Z. , Cook, P. E. , Phuc, H. K. , & Sinkins, S. P. (2009). Immune activation by life‐shortening *Wolbachia* and reduced filarial competence in mosquitoes. Science, 326, 134–136. 10.1126/science.1177531 19797660 PMC2867033

[mbo3561-bib-0039] Kolora, L. D. , Powell, C. M. , Hunter, W. , Bextine, B. , & Lauzon, C. R. (2015). Internal extracellular bacteria of *Diaphorina citri* Kuwayama (Hemiptera: Psyllidae), the Asian citrus psyllid. Current Microbiology, 70, 710–715. 10.1007/s00284-015-0774-1 25645736

[mbo3561-bib-0040] Kruse, A. , Fattah‐Hosseini, S. , Saha, S. , Johnson, R. , Warwick, E. , Sturgeon, K. , … Heck, M. C. (2017). Combining ‘omics and microscopy to visualize interactions between the Asian citrus psyllid vector and the Huanglongbing pathogen *Candidatus* Liberibacter asiaticus in the insect gut. PLoS ONE, 12, e0179531 10.1371/journal.pone.0179531 28632769 PMC5478155

[mbo3561-bib-0041] Lopes, S. A. , Frare, G. F. , Yamamoto, P. T. , Ayres, A. J. , & Barbosa, J. C. (2007). Ineffectiveness of pruning to control citrus huanglongbing caused by *Candidatus* Liberibacter americanus. European Journal of Plant Pathology, 119, 463–468. 10.1007/s10658-007-9173-1

[mbo3561-bib-0042] Macaluso, K. R. , Pornwiroon, W. , Popov, V. L. , & Foil, L. D. (2008). Identification of *Rickettsia felis* in the salivary glands of cat fleas. Vector‐Borne and Zoonotic Diseases, 8, 391–396. 10.1089/vbz.2007.0218 18399779 PMC2978049

[mbo3561-bib-0043] Mcmeniman, C. J. , Lane, R. V. , Cass, B. N. , Fong, A. W. C. , Sidhu, M. , Wang, Y. F. , & O'neill, S. L. (2009). Stable introduction of a life‐shortening *Wolbachia* infection into the mosquito *Aedes aegypti* . Science, 323, 141–144. 10.1126/science.1165326 19119237

[mbo3561-bib-0044] Min, K. T. , & Benzer, S. (1997). *Wolbachia*, normally a symbiont of *Drosophila*, can be virulent, causing degeneration and early death. Proceedings of the National Academy of Sciences of the United States of America, 94, 10792–10796. 10.1073/pnas.94.20.10792 9380712 PMC23488

[mbo3561-bib-0045] Montllor, C. B. , Maxmen, A. , & Purcell, A. H. (2002). Facultative bacterial endosymbionts benefit pea aphids *Acyrthosiphon pisum* under heat stress. Ecological Entomology, 27, 189–195. 10.1046/j.1365-2311.2002.00393.x

[mbo3561-bib-0046] Moreira, L. A. , Iturbe‐Ormaetxe, I. , Jeffery, J. A. , Lu, G. J. , Pyke, A. T. , Hedges, L. M. , … O'Neill, S. L. (2009). A *Wolbachia* symbiont in *Aedes aegypti* limits infection with dengue, *Chikungunya*, and *Plasmodium* . Cell, 139, 1268–1278. 10.1016/j.cell.2009.11.042 20064373

[mbo3561-bib-0047] Mouton, L. , Dedeine, F. , Henri, H. , Bouletreau, M. , Profizi, N. , & Vavre, F. (2004). Virulence multiple infections and regulation of symbiotic population in the *Wolbachia*‐*Asobara tabida* symbiosis. Genetics, 168, 181–189. 10.1534/genetics.104.026716 15454536 PMC1448097

[mbo3561-bib-0048] Nakabachi, A. , Shigenobu, S. , Sakazume, N. , Shiraki, T. , Hayashizaki, Y. , Carninci, P. , … Fukatsu, T. (2005). Transcriptome analysis of the aphid bacteriocyte, the symbiotic host cell that harbors an endocellular mutualistic bacterium, *Buchnera* . Proceedings of the National Academy of Sciences of the United States of America, 102, 5477–5482. 10.1073/pnas.0409034102 15800043 PMC555734

[mbo3561-bib-0049] Nakabachi, A. , Ueoka, R. , Oshima, K. , Teta, R. , Mangoni, A. , Gurgui, M. , … Fukatsu, T. (2013). Defensive bacteriome symbiont with a drastically reduced genome. Current Biology, 23, 1478–1484. 10.1016/j.cub.2013.06.027 23850282

[mbo3561-bib-0050] Nishikori, K. , Morioka, K. , Kubo, T. , & Morioka, M. (2009). Age and morph‐dependent activation of the lysosomal system and *Buchnera* degradation in aphid endosymbiosis. Journal of Insect Physiology, 55, 351–357. 10.1016/j.jinsphys.2009.01.001 19183557

[mbo3561-bib-0051] Oliver, K. M. , Degnan, P. H. , Burke, G. R. , & Moran, N. A. (2010). Facultative symbionts in aphids and the horizontal transfer of ecologically important traits. Annual Review of Entomology, 55, 247–266. 10.1146/annurev-ento-112408-085305 19728837

[mbo3561-bib-0052] Oliver, K. M. , Moran, N. A. , & Hunter, M. S. (2005). Variation in resistance to parasitism in aphids is due to symbionts not host genotype. Proceedings of the National Academy of Sciences of the United States of America, 102, 12795–12800. 10.1073/pnas.0506131102 16120675 PMC1200300

[mbo3561-bib-0053] Oliver, K. M. , Russell, J. A. , Moran, N. A. , & Hunter, M. S. (2003). Facultative bacterial symbionts in aphids confer resistance to parasitic wasps. Proceedings of the National Academy of Sciences of the United States of America, 100, 1803–1807. 10.1073/pnas.0335320100 12563031 PMC149914

[mbo3561-bib-0054] Oliver, K. M. , Smith, A. H. , & Russell, J. A. (2014). Defensive symbiosis in the real world ‐advancing ecological studies of heritable, protective bacteria in aphids and beyond. Functional Ecology, 28, 341–355. 10.1111/fec.2014.28.issue-2

[mbo3561-bib-0055] O'Neill, S. L. , Giordano, R. , Colbert, A. M. , Karr, T. L. , & Robertson, H. M. (1992). 16S rRNA phylogenetic analysis of the bacterial endosymbionts associated with cytoplasmic incompatibility in insects. Proceedings of the National Academy of Sciences of the United States of America, 89, 2699–2702. 10.1073/pnas.89.7.2699 1557375 PMC48729

[mbo3561-bib-0056] O'Neill, S. L. , Pettigrew, M. M. , Sinkins, S. P. , Braig, H. R. , Andreadis, T. G. , & Tesh, R. B. (1997). In vitrocultivation of *Wolbachia pipientis* in an *Aedes albopictus* cell line. Insect Molecular Biology, 6, 33–39. 10.1046/j.1365-2583.1997.00157.x 9013253

[mbo3561-bib-0057] Pietri, J. E. , DeBruhl, H. , & Sullivan, W. (2016). The rich somatic life of *Wolbachia* . MicrobiologyOpen, 5, 923–936. 10.1002/mbo3.2016.5.issue-6 27461737 PMC5221451

[mbo3561-bib-0059] Rio, R. V. , Wu, Y. N. , Filardo, G. , & Aksoy, S. (2006). Dynamics of multiple symbiont density regulation during host development: Tsetse fly and its microbial flora. Proceedings of the Royal Society B‐Biological Sciences, 273, 805–814. 10.1098/rspb.2005.3399 PMC1560226 16618673

[mbo3561-bib-0060] Saha, S. , Hunter, W. B. , Reese, J. , Morgan, J. K. , Marutani‐Hert, M. , Huang, H. , & Lindeberg, M. (2012). Survey of endosymbionts in the *Diaphorina citri* metagenome and assembly of a *Wolbachia* wDi draft genome. PLoS ONE, 7, e50067 10.1371/journal.pone.0050067 23166822 PMC3500351

[mbo3561-bib-0061] Sicard, M. , Dittmer, J. , Greve, P. , Bouchon, D. , & Braquart‐Varnier, C. (2014). A host as an ecosystem: *Wolbachia* coping with environmental constraints. Environmental Microbiology, 16, 3583–3607. 10.1111/emi.2014.16.issue-12 25052143

[mbo3561-bib-0062] Siozios, S. , Sapountzis, P. , Ioannidis, P. , & Bourtzis, K. (2008). *Wolbachia* symbiosis and insect immune response. Insect Science, 15, 89–100. 10.1111/ins.2008.15.issue-1

[mbo3561-bib-0063] Subandiyah, S. , Nikoh, N. , Tsuyumu, S. , Somowiyarjo, S. , & Fukatsu, T. (2000). Complex endosymbiotic microbiota of the citrus psyllid *Diaphorina citri* (Homoptera: Psylloidea). Zoological Science, 17, 983–989. 10.2108/zsj.17.983

[mbo3561-bib-0064] Tiwari, S. , Gondhalekar, A. D. , Mann, R. S. , Scharf, M. E. , & Stelinski, L. L. (2011). Characterization of five CYP4 genes from Asian citrus psyllid and their expression levels in *Candidatus* Liberibacter asiaticus‐ infected and uninfected psyllids. Insect Molecular Biology, 20, 733–744. 10.1111/imb.2011.20.issue-6 21919983

[mbo3561-bib-0065] Tortosa, P. , Charlat, S. , Labbe, P. , Dehecq, J. S. , Barre, H. , & Weill, M. (2010). *Wolbachia* age‐sex‐specific density in *Aedes albopictus*: A host evolutionary response to cytoplasmic incompatibility? PLoS ONE, 5, e9700 10.1371/journal.pone.0009700 20300514 PMC2838780

[mbo3561-bib-0066] Tsai, J. H. , & Liu, Y. H. (2000). Biology of *Diaphorina citri* (Homoptera: Psyllidae) on four host plants. Journal of Economic Entomology, 93, 1721–1725. 10.1603/0022-0493-93.6.1721 11142304

[mbo3561-bib-0067] Walker, T. , Johnson, P. H. , Moreira, L. A. , Iturbe‐Ormaetxe, I. , Frentiu, F. D. , Mcmeniman, C. J. , … Hoffmann, A. A. (2011). The wMel *Wolbachia* strain blocks dengue and invades caged *Aedes aegypti* populations. Nature, 476, U450–U101. 10.1038/nature10355 21866159

[mbo3561-bib-0068] Weeks, A. R. , Reynolds, K. T. , Hoffmann, A. A. , & Mann, H. (2002). *Wolbachia* dynamics and host effects: What has (and has not) been demonstrated? Trends in Ecology & Evolution, 17, 257–262. 10.1016/S0169-5347(02)02480-1

[mbo3561-bib-0069] Weeks, A. R. , Velten, R. , & Stouthamer, R. (2003). Incidence of a new sex‐ratio‐distorting endosymbiotic bacterium among arthropods. Proceedings of the Royal Society B‐Biological Sciences, 270, 1857–1865. 10.1098/rspb.2003.2425 PMC1691448 12964989

[mbo3561-bib-0070] Weinert, L. A. , Araujo‐Jnr, E. V. , Ahmed, M. Z. , & Welch, J. J. (2015). The incidence of bacterial endosymbionts in terrestrial arthropods. Proceedings of the Royal Society B‐Biological Sciences, 282, 20150249 10.1098/rspb.2015.0249 PMC4424649 25904667

[mbo3561-bib-0071] Werren, J. H. (1997). Biology of *Wolbachia* . Annual Review of Entomology, 42, 587–609. 10.1146/annurev.ento.42.1.587 15012323

[mbo3561-bib-0072] Werren, J. H. , Baldo, L. , & Clark, M. E. (2008). *Wolbachia*: Master manipulators of invertebrate biology. Nature Reviews Microbiology, 6, 741–751. 10.1038/nrmicro1969 18794912

[mbo3561-bib-0073] Xi, Z. Y. , & Dobson, S. L. (2005). Characterization of *Wolbachia* transfection efficiency by using microinjection of embryonic cytoplasm and embryo homogenate. Applied and Environmental Microbiology, 71, 3199–3204. 10.1128/AEM.71.6.3199-3204.2005 15933022 PMC1151837

[mbo3561-bib-0074] Yang, Y. P. , Huang, M. D. , Beattie, G. A. C. , Xia, Y. L. , Ouyang, G. C. , & Xiong, J. J. (2006). Distribution, biology, ecology and control of the psyllid *Diaphorina citri* Kuwayama, a major pest of citrus: A status report for China. International Journal of Pest Management, 52, 343–352. 10.1080/09670870600872994

[mbo3561-bib-0075] Zabalou, S. , Riegler, M. , Theodorakopoulou, M. , Stauffer, C. , Savakis, C. , & Bourtzis, K. (2004). *Wolbachia*‐induced cytoplasmic incompatibility as a means for insect pest population control. Proceedings of the National Academy of Sciences of the United States of America, 101, 15042–15045. 10.1073/pnas.0403853101 15469918 PMC524042

[mbo3561-bib-0076] Zchori‐Fein, E. , & Perlman, S. J. (2004). Distribution of the bacterial symbiont *Cardinium* in arthropods. Molecular Ecology, 13, 2009–2016. 10.1111/mec.2004.13.issue-7 15189221

[mbo3561-bib-0077] Zug, R. , & Hammerstein, P. (2012). Still a host of hosts for *Wolbachia*: Analysis of recent data suggests that 40% of terrestrial arthropod species are infected. PLoS ONE, 7, e38544 10.1371/journal.pone.0038544 22685581 PMC3369835

[mbo3561-bib-0078] Zug, R. , & Hammerstein, P. (2015a). Bad guys turned nice? A critical assessment of *Wolbachia* mutualisms in arthropod hosts. Biological Reviews, 90, 89–111. 10.1111/brv.2015.90.issue-1 24618033

[mbo3561-bib-0079] Zug, R. , & Hammerstein, P. (2015b). *Wolbachia* and the insect immune system: What reactive oxygen species can tell us about the mechanisms of *Wolbachia*‐host interactions. Frontiers in Microbiology, 6, 1201. 26579107 10.3389/fmicb.2015.01201 PMC4621438

